# Lactate-driven metabolic–epigenetic reprogramming in breast cancer: immune evasion and therapeutic resistance

**DOI:** 10.1186/s13148-026-02216-8

**Published:** 2026-07-27

**Authors:** Lei Sun, Li Wang, Wanqin Zeng, Shiyan Zeng

**Affiliations:** 1 Department of Radiation Oncology, Sichuan Clinical Research Center for Cancer, Sichuan Cancer Hospital & Institute, Sichuan Cancer Center, Affiliated Cancer Hospital of University of Electronic Science and Technology of China, Chengdu, 610041 China; 2 Department of General Internal Medicine, Sichuan Clinical Research Center for Cancer, Sichuan Cancer Hospital & Institute, Sichuan Cancer Center, Affiliated Cancer Hospital of University of Electronic Science and Technology of China, No. 55, Section 4, Renmin South Road, Wuhou District, Chengdu, 610041 China; 3 Department of Breast, Sichuan Clinical Research Center for Cancer, Sichuan Cancer Hospital & Institute, Sichuan Cancer Center, Affiliated Cancer Hospital of University of Electronic Science and Technology of China, No. 55, Section 4, Renmin South Road, Wuhou District, Chengdu, 610041 China

**Keywords:** Breast cancer, Epigenetic reprogramming, Lactate signaling, Lactylation, Metabolic reprogramming, Tumor microenvironment

## Abstract

**Background:**

Breast cancer develops within a metabolically heterogeneous tumor microenvironment characterized by regional hypoxia, glycolytic activation, lactate accumulation, extracellular acidification, and impaired antitumor immunity. Lactate is now recognized not only as a metabolic end product, but also as a transported substrate, a receptor ligand, and a potential source of lysine lactylation.

**Main body:**

In this Review, we summarize current evidence on lactate production, MCT1/MCT4-dependent trafficking, GPR81/HCAR1-mediated sensing, immune remodeling, and metabolic–epigenetic regulation in breast cancer. We organize these effects into three interconnected layers. First, extracellular acidification, nutrient competition, and redox stress produce rapid and potentially reversible suppression of CD8⁺ T cells, natural killer cells, and dendritic cells. Second, sustained lactate-rich conditions alter the relative fitness of immune and stromal populations, weakening cytotoxic effectors while supporting regulatory T cells, tumor-associated macrophages, suppressive myeloid populations, and stromal immune exclusion. Third, histone and non-histone lactylation, together with RNA-level and chromatin regulatory mechanisms, may contribute to more durable state fixation. We distinguish direct breast cancer evidence from mechanisms established in other cancers and from transcriptome-based lactylation-associated signatures. We also discuss methodological limitations, subtype-specific evidence, and therapeutic strategies targeting lactate production, transport, sensing, and downstream epigenetic regulation.

**Short conclusion:**

Lactate-driven metabolic and epigenetic regulation provides a useful framework for understanding immune evasion and therapeutic resistance in breast cancer. However, direct mechanistic evidence remains concentrated in triple-negative breast cancer and tumor-intrinsic phenotypes. Lactylation should therefore be considered a candidate mechanism of durable state fixation rather than a universal explanation for lactate-associated effects. Future studies integrating direct lactylation measurements, spatial multi-omics, functional perturbation, and patient-derived models will be required to define clinically actionable lactate-dependent states.

## Introduction

Breast cancer is a clinically and biologically heterogeneous disease, among which triple-negative breast cancer (TNBC) represents the most aggressive subtype, characterized by early recurrence, high metastatic potential, and limited response to current systemic therapies [1]. Despite the clinical success of immune checkpoint blockade in multiple malignancies, a substantial fraction of breast tumors remains resistant to immunotherapy due to insufficient immune infiltration, immunosuppressive tumor ecosystems, and profound metabolic constraints within the tumor microenvironment (TME) [2, 3]. These limitations underscore that antitumor immunity is governed not only by tumor antigenicity or immune-cell abundance, but also by the broader ecological context shaped by nutrient availability, oxygen gradients, stromal organization, and metabolite-driven signaling networks [4–6]. Among these determinants, lactate has emerged as a central metabolite linking metabolic rewiring to immune dysfunction and therapeutic resistance in breast cancer.

**Fig. 1:**
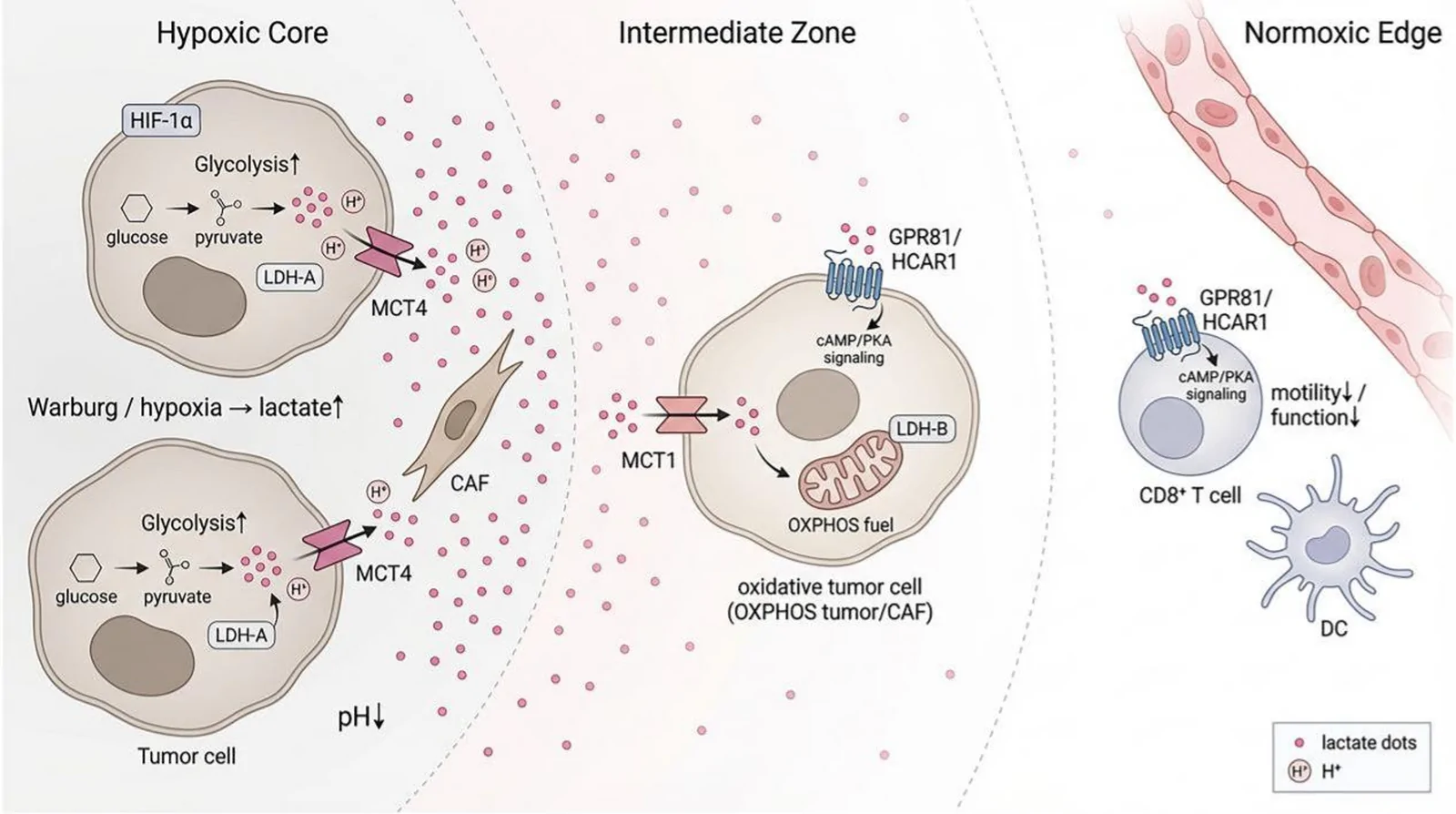
Spatial organization of lactate-rich niches in the breast cancer tumor microenvironment. In hypoxic and highly glycolytic tumor regions, HIF-1α-associated metabolic reprogramming increases LDHA-dependent lactate production. Lactate and protons are exported predominantly through MCT4, contributing to extracellular lactate accumulation and acidification. In intermediate tumor regions, MCT1-mediated lactate uptake supports oxidative metabolism in tumor or stromal cells. Extracellular lactate can also activate GPR81/HCAR1-dependent signaling. At the more normoxic tumor edge, lactate-associated signaling and metabolic stress may impair CD8⁺ T-cell motility and function and influence dendritic-cell activity. Created in BioRender. Sun, L. (2026) https://BioRender.com/2uum25v. CAF, cancer-associated fibroblast; DC, dendritic cell; GPR81/HCAR1, G protein-coupled receptor 81/hydroxycarboxylic acid receptor 1; HIF-1α, hypoxia-inducible factor 1α; LDHA, lactate dehydrogenase A; MCT, monocarboxylate transporter

Lactate was historically regarded as a terminal by-product of glycolysis; however, it is now recognized as a multifunctional regulator of tumor biology. In breast cancer, oncogenic signaling and hypoxia-driven metabolic reprogramming enhance glycolytic flux, resulting in sustained lactate accumulation and extracellular acidification, which collectively establish a metabolically hostile microenvironment that impairs cytotoxic T-cell and natural killer (NK) cell activity [5, 7, 8]. Beyond this acute metabolic stress, lactate functions as a dynamic substrate for intercellular metabolic exchange, enabling metabolic symbiosis between glycolytic tumor regions and oxidative stromal or tumor compartments [9, 10]. In addition, lactate exerts signaling functions through the GPR81/HCAR1 receptor axis, influencing tumor progression, stromal remodeling, and immune regulation [11–13]. More recently, lactate has been implicated in lysine lactylation, a post-translational modification that links cellular metabolic state to transcriptional regulation and chromatin remodeling, thereby providing a potential mechanism connecting transient metabolic stress to durable gene regulatory programs [14–16]. The spatial organization of lactate production, transport, and signaling in the breast cancer TME is illustrated in Fig. 1.

Importantly, lactate-driven regulation in breast cancer is highly context dependent and spatially heterogeneous. Tumor tissues contain hypoxic cores, invasive margins, perivascular regions, necrotic zones, and CAF-rich stromal compartments, each characterized by distinct oxygen tension, extracellular pH, lactate concentration, transporter expression, and immune-cell composition [6, 17, 18]. These spatial differences critically determine whether lactate functions primarily as a metabolic stressor, a signaling ligand, or an epigenetic modifier. Within the TME, lactate suppresses CD8 + T cells and NK cells, impairs dendritic-cell antigen presentation, and promotes regulatory T-cell and tumor-associated macrophage (TAM) expansion, collectively driving immune exclusion and tumor progression [5, 17, 19–21]. However, these effects arise through multiple mechanistically distinct processes, including extracellular acidosis, nutrient competition, receptor-mediated signaling, metabolic shuttling, and potential epigenetic modifications. Distinguishing these layers is essential for accurately interpreting lactate-associated immune dysfunction in breast cancer.

**Fig. 2:**
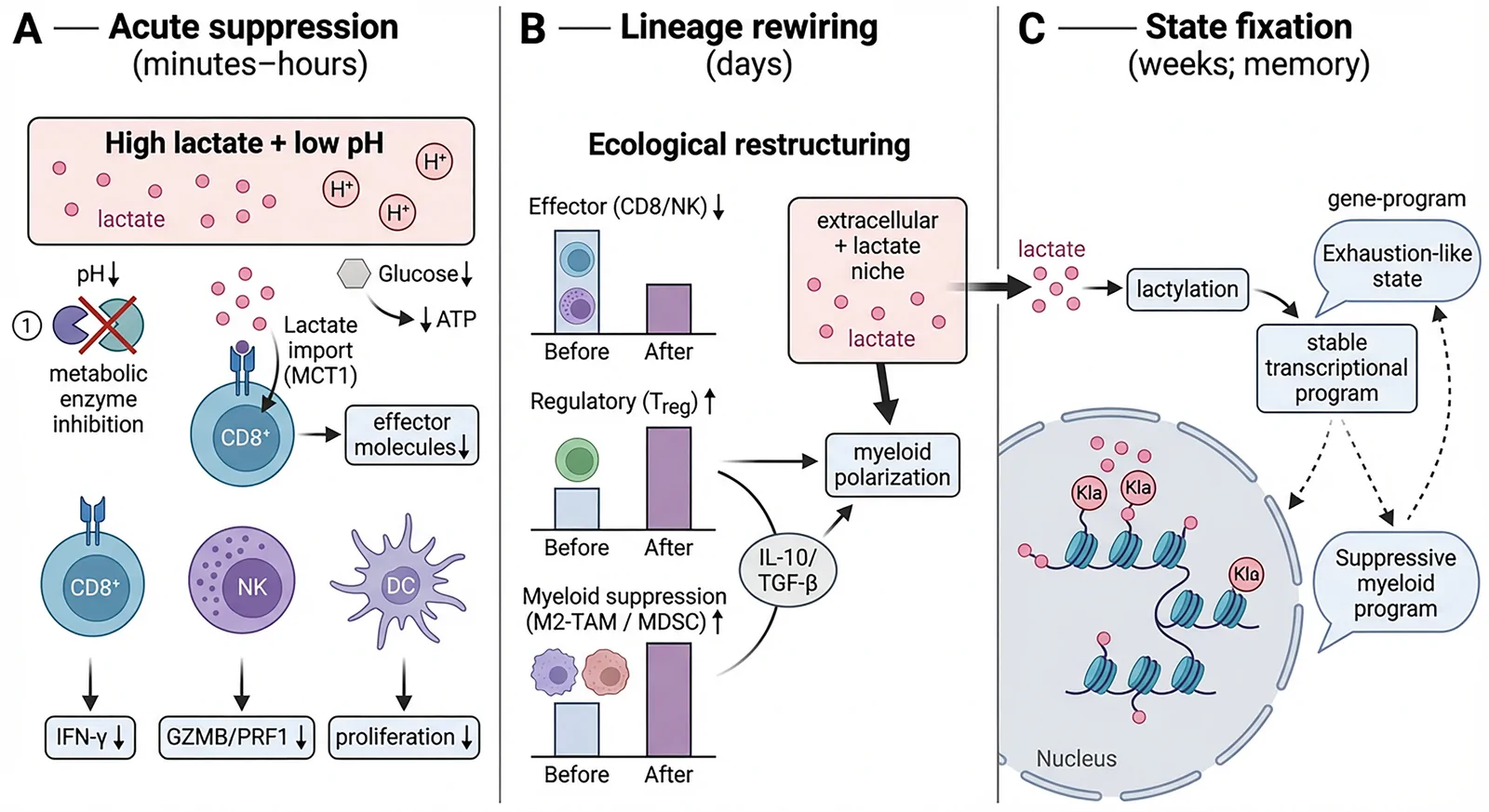
A three-layer model of lactate-driven immune regulation in breast cancer.** A** During acute metabolic suppression, high extracellular lactate, low pH, reduced glucose availability, impaired metabolic-enzyme activity, and altered lactate transport rapidly reduce CD8⁺ T-cell, natural killer-cell, and dendritic-cell function, including cytokine production, cytotoxic-molecule expression, and proliferation. **B** During lineage rewiring, sustained lactate exposure reduces the relative abundance or activity of cytotoxic effector cells while favoring regulatory T cells and suppressive myeloid populations, including M2-like tumor-associated macrophages and myeloid-derived suppressor cells. Lactate-rich niches and immunoregulatory mediators such as IL-10 and TGF-β further promote myeloid polarization. **C** During state fixation, lactate-associated lactylation may contribute to stable transcriptional programs linked to exhaustion-like lymphocyte states and suppressive myeloid programs. Created in BioRender. Sun, L. (2026) https://BioRender.com/vjnzc3k. DC, dendritic cell; GZMB, granzyme B; IFN-γ, interferon-γ; IL-10, interleukin-10; MDSC, myeloid-derived suppressor cell; NK, natural killer; PRF1, perforin 1; TAM, tumor-associated macrophage; TGF-β, transforming growth factor-β; Treg, regulatory T cell

Despite rapid progress, the mechanistic hierarchy linking lactate accumulation to immune escape remains incompletely resolved. We propose that lactate-driven regulation in breast cancer can be interpreted through three interconnected layers. The first is acute metabolic suppression, in which extracellular acidification, nutrient competition, and redox imbalance rapidly impair immune-cell signaling and effector function [5, 7, 8, 22, 23]. The second is lineage remodeling, in which sustained metabolic pressure changes the relative fitness and spatial distribution of cytotoxic, regulatory, myeloid, and stromal populations. The third is durable state fixation, in which lactylation, RNA processing, chromatin remodeling, and related epigenetic mechanisms may stabilize transcriptional or functional states after prolonged exposure [14, 16]. These layers are mechanistically related but should not be treated as interchangeable. The proposed three-layer framework is summarized in Fig. 2.

In this Review, we use this framework to distinguish well-established metabolic effects from emerging epigenetic mechanisms. We first discuss lactate production, transport, and sensing, followed by its acute and lineage-specific effects within the breast cancer TME. We then evaluate direct breast cancer evidence for histone and non-histone lactylation, mechanisms demonstrated in other tumor types, and the methodological requirements needed to establish lactylation-specific causality. Importantly, most direct mechanistic studies have been performed in TNBC or in tumor-cell-centered models, whereas evidence in hormone receptor-positive and HER2-positive breast cancer remains comparatively limited. We therefore avoid extrapolating TNBC findings to all breast cancer subtypes and conclude by considering biomarker-guided therapeutic strategies and future spatial and single-cell approaches.

## Lactate production, transport, and sensing in breast cancer

The lactate ecology of breast cancer can be understood through three interconnected layers: production, transport, and sensing. Lactate production determines the metabolic load generated by glycolytic tumor cells; lactate transport shapes where lactate accumulates and which cell populations can exchange or utilize it; and lactate sensing converts extracellular lactate availability into intracellular signaling responses. Together, these layers define the metabolic geography of the breast cancer tumor microenvironment (TME) and provide a framework for understanding how lactate influences immune-cell function, stromal organization, and lactylation-associated state fixation [13, 14, 24]. Importantly, lactate-related immune regulation should not be attributed to one mechanism alone. Lactate may act as a transported metabolite, lactic acid and proton-coupled transport may contribute to extracellular acidosis, extracellular lactate may function as a receptor ligand, and intracellular lactate may support post-translational modifications such as lysine lactylation. Distinguishing these mechanisms is essential because extracellular acidification, receptor signaling, metabolic shuttling, and intracellular lactylation require different experimental controls and are unlikely to contribute equally across all breast cancer contexts.

### Hypoxia-driven glycolysis and lactate accumulation

Hypoxia and oncogenic signaling increase glycolytic flux in breast cancer cells, in part through induction of HIF-1-related metabolic programs [25, 26]. Under these conditions, pyruvate is preferentially converted into lactate, which is exported together with protons through monocarboxylate transporters. Poor perfusion, regional oxygen gradients, and high tumor-cell density further reinforce lactate accumulation and extracellular acidification. This process generates a metabolically hostile niche in which effector lymphocytes encounter low pH, limited nutrient availability, and redox stress.

Lactate accumulation has two biologically distinct consequences. First, lactate export and proton-coupled acidification contribute to an extracellular environment that is unfavorable for cytotoxic immune cells, including CD8 + T cells and natural killer cells. Second, lactate itself can serve as a substrate or signaling input for downstream processes, including metabolic shuttling, receptor-mediated signaling, and lactate-associated post-translational modifications such as lactylation [10, 14]. Therefore, high lactate levels in breast tumors should be interpreted not only as a marker of glycolytic stress but also as a potential driver of immune suppression and durable tumor adaptation.

### MCT1/MCT4-mediated lactate trafficking and metabolic shuttling

Monocarboxylate transporters, particularly MCT1 and MCT4, are key determinants of lactate distribution in breast cancer. Rather than functioning only as transport molecules, MCT1 and MCT4 help establish spatial lactate gradients between malignant cells, stromal cells, and immune compartments. MCT4 is commonly associated with highly glycolytic and hypoxic tumor regions, where it supports lactate efflux and extracellular acidification. In contrast, MCT1 can mediate lactate uptake in more oxidative tumor or stromal compartments, allowing lactate to be used as a metabolic substrate [27–29]. This division of labor enables metabolic shuttling, in which glycolytic cells export lactate and neighboring oxidative cells import and utilize it, thereby supporting metabolic cooperation within spatially heterogeneous tumors [9, 10].

Breast cancer evidence supports the functional relevance of this axis. MCT1 expression has been associated with glycolytic phenotypes and poor prognosis, whereas functional screening studies have identified MCT4 as an important regulator of breast cancer cell metabolism and survival [28, 29]. These findings suggest that MCT1 and MCT4 are not merely markers of metabolic reprogramming but active contributors to lactate-rich tumor ecology. By regulating lactate export, uptake, and spatial compartmentalization, MCTs may influence tumor-cell fitness, extracellular acidosis, stromal interactions, and immune-cell accessibility. Therefore, MCT expression should be interpreted together with hypoxia status, extracellular pH, immune infiltration, and stromal organization rather than as an isolated metabolic feature.

### GPR81/HCAR1-dependent lactate sensing

Lactate can also act as a signaling ligand through GPR81, also known as HCAR1. GPR81/HCAR1 is a Gi-coupled lactate receptor that links extracellular lactate abundance to intracellular signaling, including cAMP-related pathways [11–13, 30]. In breast cancer models, HCAR1 has been implicated in tumor growth, migration, metabolic adaptation, and crosstalk with antigen-presenting cells in the tumor microenvironment [11, 12, 30]. These findings place GPR81 within the lactate ecology as a receptor-based mechanism through which extracellular lactate can be translated into tumor-cell, stromal-cell, and immune-cell state changes.

The immune consequences of GPR81 signaling are likely context dependent. Some breast cancer data suggest that GPR81 activation may regulate immunomodulatory phenotypes, including PD-L1-related changes in TNBC models [31]. Other studies have linked GPR81 to tumor growth, invasiveness, extracellular matrix remodeling, and Notch ligand regulation [11, 18]. Therefore, GPR81 should not be described as a uniformly immunosuppressive or universally targetable pathway. Its biological output probably depends on the receptor-expressing cell type, including malignant cells, antigen-presenting cells, endothelial cells, CAFs, and other stromal compartments. Future studies should define GPR81 signaling in a cell-type-specific and spatially resolved manner, with attention to whether its dominant effect involves tumor proliferation, antigen-presenting-cell function, checkpoint regulation, extracellular matrix remodeling, or immune-cell recruitment.

## Acute metabolic suppression and immune-lineage remodeling in the breast cancer TME

Lactate-rich breast tumors suppress antitumor immunity through both immediate metabolic stress and longer-term immune-lineage remodeling. Acute suppression is mainly driven by extracellular acidification, nutrient competition, and redox stress, whereas sustained lactate exposure can reshape the relative fitness of immune populations and promote a more stable immunosuppressive ecology. Therefore, lactate should not be viewed simply as a factor that globally inhibits immune cells. Instead, lactate-rich niches selectively weaken cytotoxic effector cells while supporting regulatory, myeloid, and stromal programs that contribute to immune exclusion and therapeutic resistance [17, 19, 20]. The major lineage-specific effects of lactate and their current evidence status are summarized in Table 1.

**Table 1:** Lactate-driven immune modulation across major lineages in the TME

Cellular compartment	Major lactate-related input	Dominant functional consequence	Evidence status
CD8⁺ T cells	High lactate, low pH, glucose limitation; sustained lactate metabolism	Reduced proliferation, cytokine production, cytotoxicity, and infiltration; possible persistent dysfunction	Strong pan-cancer metabolic evidence; limited breast cancer-specific lactylation evidence
NK cells	Lactate-associated acidification and metabolic stress	Reduced cytotoxic activity and IFN-γ production	Established metabolic effect; limited evidence for durable epigenetic fixation
Dendritic cells	Lactate, low pH, lactate–SREBP2 signaling	Impaired maturation, antigen presentation, and cross-priming	Mechanistically supported across tumor models; breast cancer-specific validation remains limited
Tregs	Lactate-rich, glucose-poor conditions; RNA-splicing regulation	Relative metabolic fitness and preserved suppressive function; increased CTLA-4/PD-1/TNFR2-related programs in selected contexts	Strong tumor-context evidence; not all mechanisms validated in breast cancer
TAMs and suppressive myeloid cells	Lactate uptake, ERK/STAT3 signaling, mitochondrial metabolism, candidate lactylation	Immunosuppressive polarization, tissue remodeling, angiogenesis, and T-cell suppression	Direct breast cancer evidence for TAM regulation; MDSC and lactylation-specific evidence remains emerging
CAF/ECM compartments	Lactate gradients, GPR81-associated signaling, hypoxia, matrix organization	Spatial immune exclusion and restricted T-cell/DC access	Strong spatial rationale; direct lactylation-dependent evidence is limited

### Acute metabolic suppression by acidosis and nutrient competition

The most immediate immune effect of lactate-rich tumors is biochemical. Lactate export is coupled to proton transport, resulting in extracellular acidification [7, 8, 22]. Low extracellular pH can impair T-cell receptor signaling, cytokine production, cytotoxic granule release, survival, and migratory capacity of effector lymphocytes. NK cells and dendritic cells are also sensitive to acidic stress, which can reduce cytotoxic activity and weaken antigen-priming capacity [22, 23]. These effects do not necessarily require stable epigenetic reprogramming; rather, they can occur rapidly when immune cells encounter a hostile extracellular environment.

Nutrient competition further reinforces this suppression. Highly glycolytic breast cancer cells consume glucose and reshape local availability of amino acids, lipids, and redox-active metabolites [32, 33]. Activated CD8^+^ T cells also depend on glycolysis to proliferate and generate effector molecules. When glucose becomes limited while lactate and acidity accumulate, effector cells experience simultaneous substrate deprivation, pH stress, and redox imbalance. This metabolic constraint reduces their ability to expand, survive, infiltrate the tumor bed, and deliver cytotoxic molecules at sufficient intensity. As a result, lactate-rich tumors may become less responsive to immune checkpoint blockade because fewer T cells remain metabolically competent enough to be effectively reinvigorated.

These effects define the acute metabolic layer of lactate-driven immune suppression. They can occur before stable transcriptional or chromatin changes are established and may remain partly reversible when extracellular pH, nutrient availability, or lactate transport is corrected. Accordingly, acute dysfunction should be experimentally separated from longer-term lineage remodeling and candidate epigenetic state fixation [14, 16].

### CD8^+^ T cells and NK cells

CD8 + T cells and NK cells are the principal cytotoxic effectors of antitumor immunity but are highly sensitive to lactate-associated acidosis and metabolic stress. Acute exposure to elevated lactate and low pH reduces proliferation, cytokine production, cytotoxic granule release, and tumor infiltration [5, 22], thereby directly impairing tumor cell elimination. This vulnerability is particularly relevant in breast cancer, especially triple-negative subtypes, where glycolytic metabolism and hypoxia frequently coexist with limited responsiveness to immunotherapy.

Beyond acute dysfunction, sustained exposure to lactate-rich conditions may contribute to more persistent metabolic impairment and exhaustion-like states. In a recent study outside the breast cancer setting, MCT11, encoded by SLC16A11, was shown to enforce dysfunction in exhausted T cells by supporting lactate metabolism [34]. This finding should not be confused with the better-characterized MCT1 transporter and should currently be interpreted as a transferable mechanism rather than a breast cancer-specific pathway. Therapeutically, transient dysfunction caused by acidosis or nutrient limitation may be partly reversible, whereas prolonged dysfunction may require combined correction of the metabolic environment and downstream immune or epigenetic programs [34, 35].

### Dendritic cells and antigen priming

Dendritic cells are essential for linking tumor antigen availability to effective CD8^+^ T-cell priming. Lactate-rich and acidic microenvironments impair dendritic-cell maturation, antigen presentation, and cross-priming capacity. Mechanistically, lactate can induce tolerogenic dendritic-cell programs through an SREBP2-associated pathway, resulting in reduced CD8^+^ T-cell priming [21]. This provides a mechanistic model through which antigen availability may fail to generate effective cytotoxic immunity when dendritic-cell maturation and cross-priming are metabolically impaired.

Importantly, dendritic-cell dysfunction represents an upstream regulatory event that can limit effector immune activation even in the presence of sufficient antigenic signals. Thus, lactate-mediated suppression of dendritic-cell function contributes to immune escape by preventing proper initiation of adaptive immune responses in the breast cancer tumor immune microenvironment.

### Regulatory T cells

Regulatory T cells (Tregs) are comparatively well adapted to lactate-rich and glucose-limited tumor environments. Unlike activated effector T cells, which depend heavily on glycolysis, tumor-infiltrating Tregs can use lactic acid-derived carbon to support oxidative metabolism and maintain suppressive activity under nutrient stress [19]. Lactate may therefore create a relative fitness advantage for Tregs even when it does not directly increase their abundance.

Additional mechanisms have been reported in different tumor contexts. Lactate-dependent regulation of RNA splicing can increase CTLA-4 expression in tumor-infiltrating Tregs, while lactate-associated programs have also been linked to PD-1 and TNFR2 expression [36–38]. However, these mechanisms have not all been demonstrated directly in breast cancer. They should therefore be presented as biologically relevant models that require validation in breast cancer subtypes and patient samples. Overall, lactate-rich conditions may shift the balance of the T-cell compartment by simultaneously impairing cytotoxic effectors and preserving regulatory-cell function.

### TAMs and suppressive myeloid reprogramming

Tumor-associated macrophages (TAMs) are highly responsive to lactate-rich tumor niches and can adapt their metabolic state to support immunosuppressive functions [20, 39–41]. In breast cancer, tumor-derived lactate has been linked to M2-like macrophage polarization via ERK/STAT3 signaling, while lactate-dependent mitochondrial metabolism and ACLY-associated histone acetylation further support macrophage differentiation programs [20, 40, 41]. Lactylation may provide an additional regulatory layer contributing to stabilization of macrophage transcriptional states, although its functional relevance in breast cancer TAMs requires further validation [14, 17].

Functionally, lactate-conditioned TAMs can suppress CD8⁺ T-cell activity, impair dendritic-cell priming, support regulatory-cell recruitment, and produce mediators involved in angiogenesis, invasion, and extracellular matrix remodeling [42, 43]. Increased TAM abundance and M2-like features are associated with aggressive breast cancer phenotypes, although macrophage polarization cannot be reduced to a single binary state [43]. Other suppressive myeloid populations, including MDSC-like states, may also be favored in hypoxic and poorly perfused regions. However, direct breast cancer evidence connecting lactate or lactylation to defined MDSC programs remains limited, and this part of the model should be presented as an emerging rather than established mechanism [17, 42].

### CAF/ECM-associated spatial immune exclusion

The immune effect of lactate is also spatial. Breast tumors contain hypoxic tumor nests, invasive fronts, perivascular regions, necrotic areas, CAF-rich stromal zones, and immune-cell clusters. These compartments may differ in lactate concentration, extracellular pH, oxygen tension, MCT expression, immune-cell access, and stromal organization. CAFs and extracellular matrix barriers can restrict T-cell and dendritic-cell entry into malignant-cell regions, while lactate-rich niches may further favor macrophages, immature myeloid cells, and Tregs [17–20].

Lactate may strengthen spatial immune exclusion through several non-mutually exclusive mechanisms. It can maintain glycolytic tumor regions, promote myeloid and regulatory immune dominance, and potentially influence extracellular matrix remodeling through GPR81-dependent or stromal-associated signaling pathways [11, 18]. Therefore, lactate-driven immune evasion should be understood not only as a cell-intrinsic metabolic process but also as a tissue-level spatial circuit. This perspective is important for therapy because metabolic correction alone may be insufficient if effector cells remain physically excluded from tumor nests by CAF/ECM barriers.

Taken together, lactate-rich breast tumors create an immune-suppressive circuit in which acidosis and nutrient competition rapidly impair effector cells, while sustained lactate exposure supports Tregs, TAMs, suppressive myeloid populations, and stromal barriers. This layered mechanism provides a conceptual bridge between acute metabolic suppression, immune-lineage remodeling, spatial exclusion, and resistance to immunotherapy.

## Lactylation and Candidate Metabolic–Epigenetic State Fixation

Whether lactylation functions as a causal driver, a permissive regulatory layer, or a correlate of lactate accumulation remains context dependent. The modification provides a plausible route through which sustained metabolic activity may influence chromatin regulation and protein function, but lactate exposure also alters extracellular pH, redox balance, substrate availability, receptor signaling, and multiple acyl-CoA pools. Lactylation should therefore not be used as a default explanation for every phenotype observed under high-lactate conditions. In this section, we distinguish the molecular machinery of lactylation, direct breast cancer evidence, mechanisms established in other cancers, RNA-level and non-histone regulation, and the experimental criteria required to establish lactylation-specific causality.

### Enzymatic and non-enzymatic regulation of lysine lactylation

Lysine lactylation is a lactate-associated post-translational modification that can occur on histone and non-histone proteins [14, 15, 44–47]. Histone lactylation may influence transcription and chromatin accessibility, whereas non-histone lactylation can alter protein stability, localization, enzymatic activity, molecular interactions, RNA processing, and signaling output. Lactylation can arise through more than one biochemical route, including enzyme-dependent L-lactylation and non-enzymatic lactoylation mediated by reactive lactoylglutathione-derived intermediates [45, 48, 49].

Several proteins have been proposed to install lactylation. p300/CBP, HBO1, KAT8, AARS1, and AARS2 have been implicated as lactyltransferases in specific experimental contexts [14, 50–53]. Recent evidence further indicates that class I histone deacetylases HDAC1, HDAC2, and HDAC3 can catalyze lysine lactylation in a lactate-concentration-dependent and reversible reaction [54]. This finding complicates a simple writer–eraser classification because the same enzymes may catalyze either the addition or removal of lactyl groups depending on substrate availability and reaction conditions. Class I HDACs and several sirtuins have also been reported to remove lactylation marks [55, 56].

**Fig. 3:**
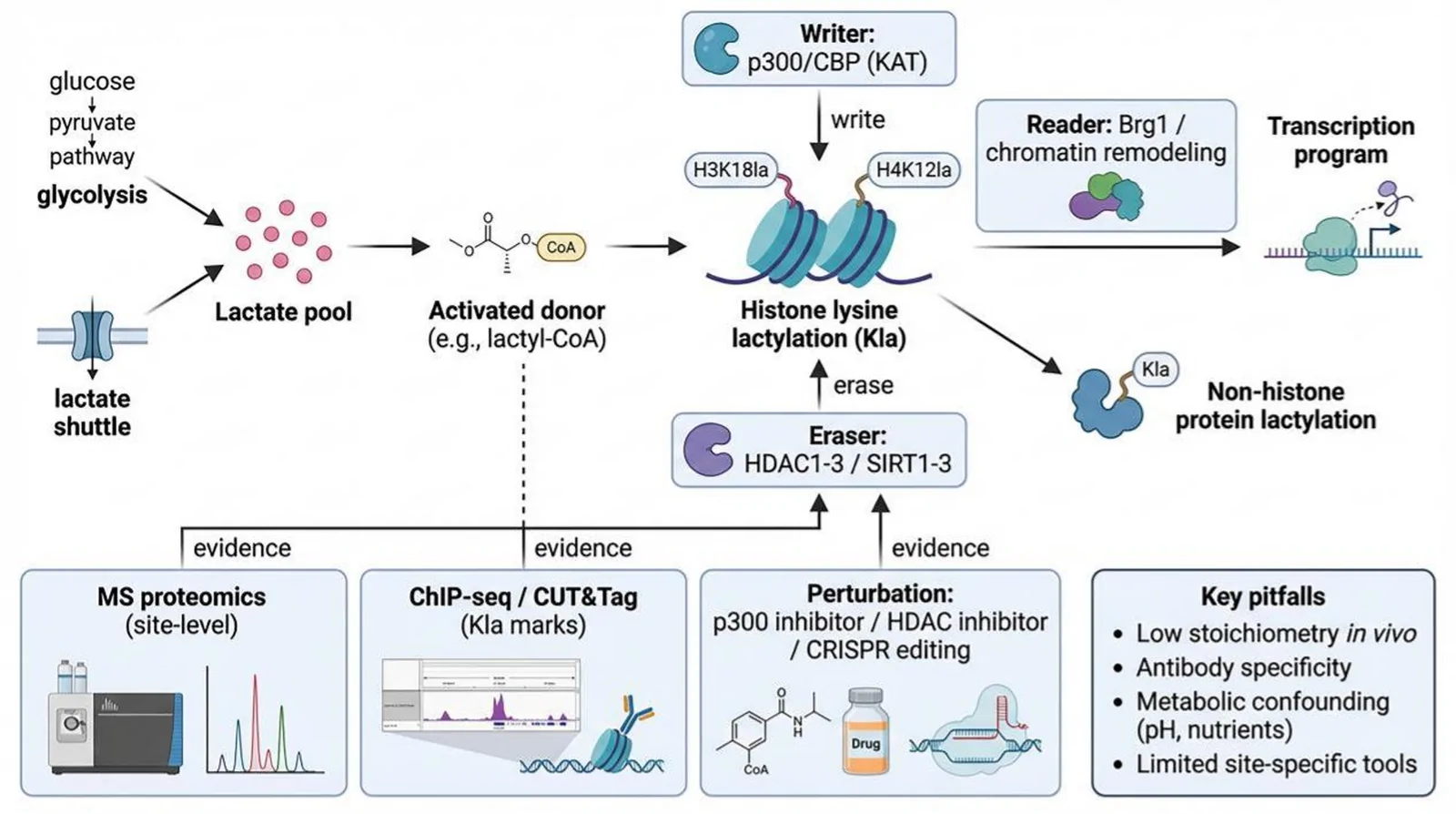
Molecular regulation of lysine lactylation and requirements for causal validation. Glycolysis and lactate shuttling increase the intracellular lactate pool, which may contribute to the formation of activated lactyl donors and subsequent histone lysine lactylation. p300/CBP is shown as a candidate lactylation writer, whereas HDAC1–3 and SIRT1–3 are shown as candidate erasers. BRG1-mediated chromatin remodeling may interpret histone lactylation and connect this modification to downstream transcriptional programs. Lactylation may also occur on non-histone proteins. Experimental evidence can be obtained through site-resolved mass spectrometry, ChIP-seq or CUT&Tag analysis of lactylation-associated chromatin marks, and genetic or pharmacological perturbation of candidate regulatory enzymes. Important limitations include low modification stoichiometry in vivo, antibody specificity, metabolic confounding by pH and nutrient availability, and the limited availability of site-specific tools. Created in BioRender. Sun, L. (2026) https://BioRender.com/vjnzc3k. BRG1, Brahma-related gene 1; ChIP-seq, chromatin immunoprecipitation sequencing; CUT&Tag, cleavage under targets and tagmentation; HDAC, histone deacetylase; Kla, lysine lactylation; MS, mass spectrometry; SIRT, sirtuin

Candidate lactylation readers are also emerging. BRG1, TRIM33, and DPF2 can recognize or functionally interpret lactylated lysine residues in defined biological systems [57–59]. However, their relevance to breast cancer and antitumor immunity has not been established uniformly. In addition, many proposed writers, erasers, and readers also regulate acetylation or other acyl modifications. Genetic or pharmacological perturbation of these proteins therefore cannot, by itself, demonstrate that a phenotype is specifically mediated by lactylation. An overview of the proposed lactylation regulatory machinery and the experimental approaches used to assess causality is provided in Fig. 3.

### Direct breast cancer evidence and subtype distribution

Direct evidence linking lactylation to breast cancer progression is increasing but remains concentrated in tumor-intrinsic phenotypes and TNBC-oriented models. H4K12 lactylation has been linked to TNBC progression through suppression of SLFN5, and lactyl-proteomic analysis has identified H4K12la as a potential TNBC-associated biomarker [60, 61]. H3K18 lactylation has also been implicated in PPARD-dependent survival programs and lactate-dependent c-Myc expression [62, 63]. In addition, KCNK1 was reported to increase LDHA activity, lactate production, and H3K18 lactylation, establishing a positive-feedback program that promoted breast cancer proliferation and metastasis [64].

More recent studies have extended this evidence. A SIN1–TTK–LDHA pathway was shown to increase LDHA Tyr239 phosphorylation, lactate production, H3K18la enrichment at the SLC2A3/GLUT3 promoter, and glucose uptake, thereby generating a metabolic–epigenetic feed-forward loop in hyperglycolytic breast cancer [65]. Non-histone lactylation may also contribute to metastatic behavior: LDHA-dependent lactylation of CREB1 at K136 was reported to enhance transcription of cytoskeletal remodeling genes and promote breast cancer brain metastasis [66]. These studies support direct roles for both histone and non-histone lactylation in malignant-cell behavior, but they do not yet establish that the same mechanisms drive immune evasion.

A recent single-cell integrative study identified lactylation-associated transcriptional states across ER-positive, HER2-positive, and triple-negative breast cancers, with relatively high scores in malignant epithelial cells and marked subtype heterogeneity [67]. Importantly, the score was derived from gene expression rather than direct measurement of protein lactylation. It should therefore be interpreted as a transcriptomic surrogate of lactylation-associated biology, not as evidence that lactylation itself was increased in each cell population.

Overall, breast cancer evidence is currently strongest for tumor-cell proliferation, survival, metabolic adaptation, and metastasis. Direct causal evidence linking defined lactylation sites to antigen presentation, checkpoint regulation, macrophage polarization, Treg function, or CD8⁺ T-cell exclusion remains limited. Moreover, the available mechanistic literature is weighted toward TNBC. Findings from TNBC should not be assumed to apply equally to hormone receptor-positive or HER2-positive disease without subtype-specific validation.

### Pan-cancer immune-evasion evidence relevant to breast cancer

Several lactylation-associated immune-evasion mechanisms have been described more clearly in non-breast cancer contexts. Histone lactylation has been linked to increased expression of inhibitory ligands such as PD-L1 and B7-H3 in certain tumor settings [68–70]. Lactylation-related programs may also reinforce suppressive myeloid states, immune exclusion, and therapeutic resistance in selected tumor contexts [17, 71]. In addition, lactate-linked epigenetic regulation has been associated with impaired antigen processing or MHC-I presentation, which could reduce tumor-cell visibility to CD8^+^ T cells [11, 72, 73].

These findings provide biologically plausible mechanisms that may be relevant to breast cancer, but their evidentiary status should remain explicit. Mechanisms demonstrated in ovarian cancer, hematologic malignancies, pancreatic cancer, or small-cell lung cancer should be presented as transferable hypotheses rather than established breast cancer pathways. At present, lactylation can reasonably be proposed as a candidate link between glycolytic metabolism and immune-evasive transcriptional states, but breast cancer-specific functional validation in immune-competent models and patient specimens is still required.

### RNA-level regulation and non-histone lactylation

An additional emerging question is whether lactate and lactylation regulate immune evasion through RNA-level mechanisms. Lactate has been reported to modulate RNA splicing in tumor-infiltrating regulatory T cells, thereby promoting CTLA-4 expression [36]. This finding suggests that lactate-associated immune regulation may extend beyond chromatin modification to include RNA processing, RNA-binding proteins, and non-histone lactylation.

For breast cancer, this area should currently be framed as an emerging direction rather than a definitive mechanism. Important future questions include whether lactate regulates immune-cell-specific splicing programs in breast tumors, whether lactylation modifies RNA-binding proteins, and whether RNA-level regulation cooperates with chromatin lactylation to stabilize Treg, myeloid, or exhausted T-cell states. Because direct breast cancer evidence remains limited, these possibilities should be discussed cautiously and not used as major mechanistic claims. The recently identified CREB1 K136 lactylation mechanism in breast cancer brain metastasis further illustrates how non-histone lactylation may directly alter transcription-factor activity, although its relationship to antitumor immunity remains unknown [66].

### Methodological limitations and criteria for causal inference

Several methodological limitations complicate the interpretation of lactylation studies. An increase in pan-Kla or site-specific antibody signal does not by itself establish the identity of the modified residue, distinguish L-lactylation from D-lactylation or other isobaric modifications, or quantify site occupancy [45–47, 74]. Lactyllysine and carboxyethyllysine can be difficult to distinguish by conventional LC–MS/MS because they produce the same nominal mass shift [74]. Moreover, L- and D-lactylated peptides may require synthetic standards, chromatographic separation, and targeted mass-spectrometric analysis for confident assignment [75]. Therefore, Western blotting, immunohistochemistry, or immunofluorescence should be complemented by site-resolved mass spectrometry when a specific lactylation site is central to a causal claim [49, 74, 75].

Extracellular lactate treatment simultaneously changes extracellular pH [7, 8, 22, 23], carbon and redox metabolism [24, 26, 32, 33], receptor-mediated signaling [11–13], and the metabolic conditions that support lactylation [14, 44, 49, 54]. A phenotype observed after lactate exposure therefore cannot automatically be attributed to lactylation. In addition, p300/CBP, HDACs, sirtuins, and other candidate lactylation regulators have broad roles in acetylation, deacetylation, transcription, and metabolism [14, 44, 54–56]. Their perturbation may consequently generate lactylation-independent effects. Finally, breast cancer studies have primarily linked defined lactylation events to malignant-cell proliferation, survival, and metastasis [60–67], whereas direct causal evidence in breast cancer immune and stromal populations remains limited.

Stronger causal inference requires independent control of extracellular pH [7, 8], isotope tracing and site-resolved mass spectrometry [49, 74, 75], and site-specific lysine mutation with functional rescue when a defined residue is proposed to mediate the phenotype [60, 64–66]. These approaches should be complemented by immune-competent, patient-derived, or spatially resolved models. Expression-based lactylation scores should be described as transcriptomic surrogates rather than direct measurements of protein lactylation [67].

### Crosstalk with other epigenetic mechanisms

Lactylation is unlikely to operate as an isolated epigenetic switch. Lactate accumulation alters glycolytic flux, redox balance, mitochondrial metabolism, and acyl-donor availability [24, 26, 32, 33]. Lactylation and acetylation can converge on shared regulatory enzymes, including p300/CBP and class I HDACs [14, 54, 55]. These shared regulators provide a mechanistic basis for crosstalk between lactylation and the broader acylation landscape, although direct competition between individual modifications remains site and context dependent.

Lactate-associated H4K5 lactylation has been linked to PRC2-dependent repression of antigen-presentation genes [73]. Separately, BRG1 [57] and DPF2 [59] have been identified as candidate readers linking lactylated residues to chromatin regulation.

Drugs targeting p300, HDACs, sirtuins, DNMTs, or chromatin remodelers are not selective lactylation inhibitors and may affect multiple transcriptional and metabolic pathways [14, 44–47, 54–56].

## Integrated Lineage and Spatial Circuit Logic

Lactate-rich tumor regions impose a selective pressure rather than a uniform inhibitory effect on all cell populations. Cytotoxic lymphocytes and dendritic cells are highly sensitive to extracellular acidification, nutrient scarcity, and redox imbalance, whereas Tregs, TAMs, and some suppressive myeloid states can maintain function or acquire a relative fitness advantage under the same conditions [17, 19–21, 36, 39–41]. The resulting immune landscape is therefore shaped not only by direct suppression of individual cells, but also by changes in the relative persistence and functional dominance of different lineages.

This lineage imbalance is reinforced by spatial organization. Hypoxic tumor nests, poorly perfused regions, and CAF-rich stromal compartments can accumulate lactate while restricting immune-cell access. MCT expression, extracellular pH, vascular structure, ECM density, and chemokine gradients together determine whether cytotoxic lymphocytes reach malignant-cell regions or remain confined to peripheral stromal zones. TAMs, Tregs, and immature myeloid populations may consequently become enriched in lactate-rich or stromal niches, whereas CD8⁺ T cells and dendritic cells are metabolically and physically excluded [11, 17, 18, 71].

The transition from acute suppression to a more persistent immune-evasive ecology is likely to involve several mechanisms. Repeated metabolic stress can maintain lineage imbalance and spatial exclusion even without a stable epigenetic change. Lactylation, RNA processing, histone acetylation, methylation, and chromatin remodeling may provide additional mechanisms through which prolonged exposure stabilizes selected transcriptional states. However, the relative contribution of each mechanism remains cell-type and context dependent. Durable state fixation should therefore be viewed as a testable model that integrates metabolic history, lineage selection, and spatial organization rather than as a consequence of lactylation alone.

This integrated framework has therapeutic implications. Lowering lactate production may relieve part of the acute metabolic pressure, but it may not restore antitumor immunity when effector cells remain spatially excluded or when suppressive transcriptional states have already been established. Effective treatment may therefore require combinations that address the dominant defect in each tumor: lactate production or transport, receptor-mediated signaling, stromal exclusion, persistent immune dysfunction, or candidate epigenetic state fixation.

## Therapeutic translation, limitations, and biomarker-guided strategies

Therapeutic targeting of the lactate axis can be considered at four levels: lactate production, membrane transport, receptor-mediated sensing, and downstream state maintenance. Each approach has a different biological rationale and a distinct set of limitations (Table 2). Because lactate is also used by normal tissues and activated immune cells, successful translation will require greater selectivity than can be achieved by broadly suppressing glycolysis.

**Table 2:** Therapeutic targeting of the lactate axis: tiers, logic, combinations, and practical readouts

Target level	Representative strategy	Mechanistic rationale	Development stage/evidence	Major limitations	Candidate biomarkers
Glycolysis/lactate production	2-deoxy-D-glucose; LDHA-directed strategies	Reduce glycolytic flux, lactate generation, and extracellular acidification	2-DG evaluated in phase I studies; most LDHA-selective approaches remain preclinical	Systemic metabolic toxicity; compensation through oxidative phosphorylation, glutamine, or lipid metabolism; possible impairment of activated immune cells	FDG uptake; lactate concentration; LDHA expression; hypoxia signatures
MCT1-dependent transport	AZD3965	Restrict lactate uptake and metabolic symbiosis in MCT1-dependent tumors	Phase I testing in advanced cancers; no established breast cancer efficacy	MCT4-mediated compensation; MCT1 expression in normal and immune cells; retinal and metabolic effects	MCT1/MCT4 ratio; tumor lactate uptake; oxidative phenotype
MCT4-dependent export	MSC-4381 or genetic MCT4 inhibition	Reduce lactate export and extracellular acidification in highly glycolytic tumors	Preclinical evidence, including improved checkpoint blockade in colorectal cancer models	No established breast cancer clinical evidence; possible transporter redundancy; tumor heterogeneity	MCT4 expression; glycolytic/hypoxia signatures; extracellular lactate and pH
GPR81/HCAR1 sensing	Genetic inhibition or experimental receptor antagonism	Decouple extracellular lactate from receptor-mediated tumor, stromal, or immune signaling	Preclinical	Cell-type-dependent and potentially opposing effects; lack of mature clinical inhibitors	GPR81 expression; receptor-cell compartment; cAMP-related pharmacodynamic markers
Lactylation machinery	Experimental targeting of candidate writers, erasers, or readers	Disrupt lactylation-associated transcriptional or protein states	Exploratory/preclinical; no selective clinically validated lactylation inhibitor	Poor specificity; simultaneous effects on acetylation, chromatin, metabolism, and immune cells	Site-specific Kla measured by mass spectrometry; writer/eraser expression; lactate flux
Broad epigenetic priming	DNMT or HDAC inhibitors combined with immune checkpoint blockade	Restore antigen presentation or inflammatory transcription before immunotherapy	Early clinical studies in solid tumors, but not lactylation-specific	Hematologic and immune toxicity; broad chromatin effects; uncertain treatment sequence	Antigen-presentation genes; DNA methylation; chromatin accessibility; immune infiltration
Biomarker-guided combination therapy	Lactate-axis inhibition plus ICI, stromal modulation, or short-course epigenetic priming	Match treatment to metabolic suppression, spatial exclusion, or persistent state maintenance	Mechanism-guided and largely preclinical	Need for serial sampling and validated patient-selection criteria	Lactate/pH, MCTs, hypoxia, TAM/Treg abundance, CD8⁺ localization, defined Kla sites

The first strategy is to reduce lactate production. Inhibiting glycolysis or LDHA can decrease lactate generation and extracellular acidification, but systemic metabolic toxicity and pathway compensation remain major concerns [76, 77]. Cancer cells may increase oxidative phosphorylation, glutamine use, fatty-acid metabolism, or alternative carbon flux when glycolysis is restricted. Activated T cells also require metabolic flexibility, creating a risk that poorly timed or intensive glycolytic inhibition could impair antitumor immunity. Upstream metabolic inhibition is therefore more likely to be useful in biomarker-selected, highly glycolytic tumors than as an unselected long-term treatment.

A second strategy is to block lactate transport. AZD3965, an oral MCT1 inhibitor, has completed phase I dose-escalation testing in patients with advanced cancers, demonstrating clinical feasibility but not breast cancer-specific efficacy [35]. MCT1 blockade may be most effective in tumors that depend on lactate uptake and have limited MCT4-mediated compensation. In contrast, selective MCT4 inhibition targets lactate export from highly glycolytic cells. Preclinical studies using the MCT4 inhibitor MSC-4381 have shown improved T-cell function and enhanced checkpoint blockade in colorectal cancer models [78]. These findings support the therapeutic principle but should not be presented as direct evidence in breast cancer. In addition, MCT expression in malignant and immune cells should be considered because transporter inhibition may alter both tumor metabolism and lymphocyte fitness.

A third strategy is to interrupt lactate sensing through GPR81/HCAR1. This approach may reduce receptor-mediated signaling even when extracellular lactate remains abundant [11, 13, 79]. However, GPR81 has context-dependent functions in malignant cells, antigen-presenting cells, endothelial cells, and stromal populations. Mature clinical-grade GPR81 inhibitors and validated patient-selection biomarkers are not yet available. Pharmacodynamic studies should therefore determine which cell compartment is affected rather than relying only on tumor-volume changes.

The fourth and least mature strategy is to reverse downstream state maintenance. No selective inhibitor of histone or non-histone lactylation has been clinically validated. Candidate writers, erasers, and readers also regulate acetylation, chromatin structure, transcription, and metabolism, making it difficult to attribute drug effects specifically to lactylation. Broader epigenetic agents, particularly DNA hypomethylating agents, can alter tumor immunogenicity and have been combined with checkpoint blockade in early clinical studies [80, 81]. However, these agents also affect normal immune cells, hematopoiesis, and stromal compartments. Dose, sequence, and treatment duration are therefore likely to determine whether epigenetic intervention enhances immunity or produces toxicity.

**Fig. 4:**
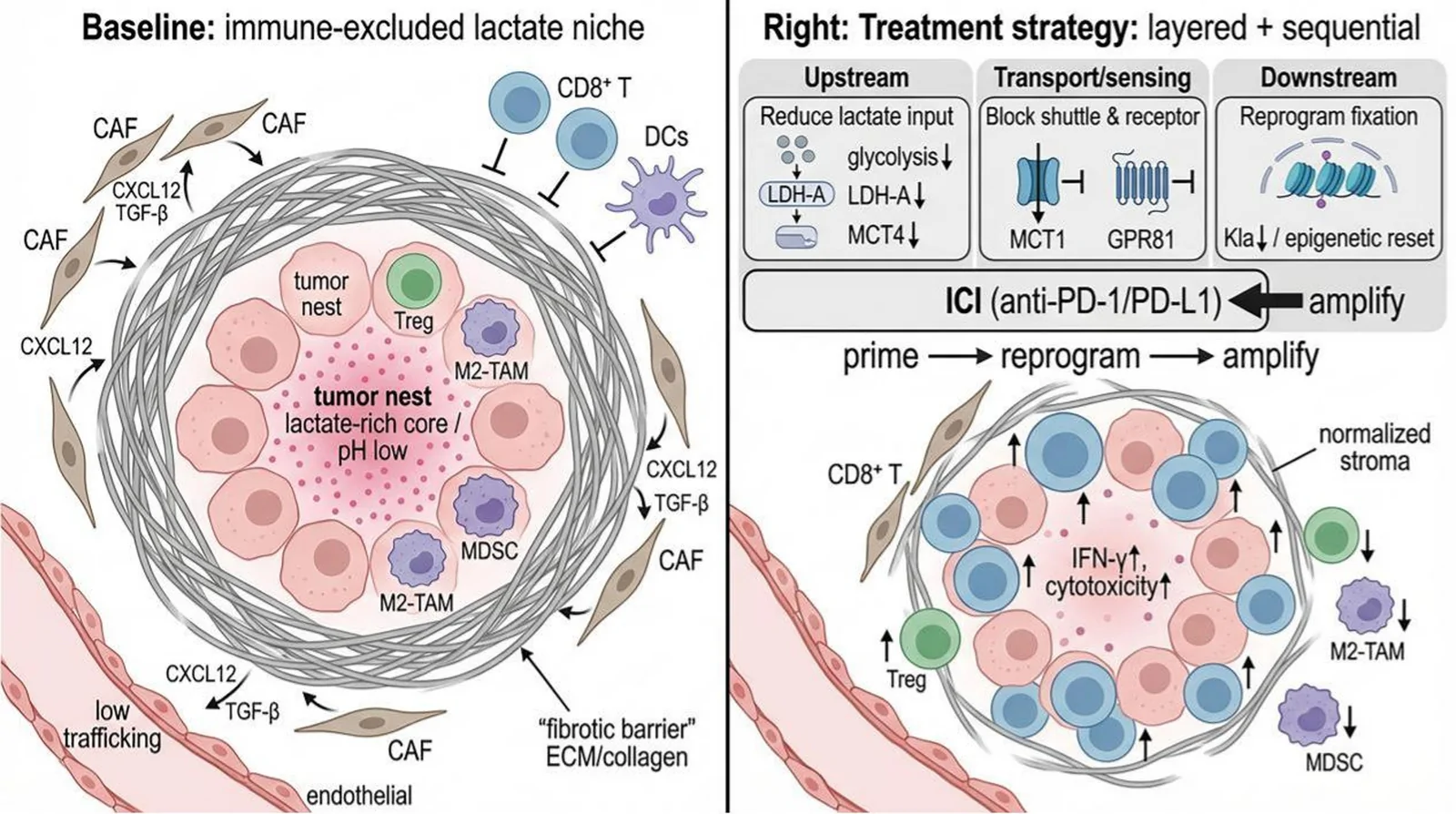
Spatial immune exclusion in lactate-rich tumors and a proposed biomarker-guided intervention framework. The left panel illustrates an immune-excluded lactate-rich tumor niche characterized by a lactate-rich, low-pH tumor core, CAF- and extracellular matrix-associated barriers, reduced immune-cell trafficking, accumulation of regulatory T cells, M2-like tumor-associated macrophages, and myeloid-derived suppressor cells, and limited access of CD8⁺ T cells and dendritic cells to the tumor nest. The right panel presents a proposed layered and sequential treatment strategy. Upstream interventions reduce lactate production, transport- and sensing-directed interventions block MCT1- or GPR81-dependent signaling, and downstream interventions aim to reverse lactate-associated epigenetic state fixation. These approaches may prime and reprogram the tumor microenvironment before amplification of antitumor immunity with immune-checkpoint blockade, resulting in improved CD8⁺ T-cell infiltration and cytotoxicity, reduced suppressive myeloid populations, and partial normalization of the stromal compartment. This treatment sequence is conceptual and has not been clinically validated as a fixed therapeutic regimen. Created in BioRender. Sun, L. (2026) https://BioRender.com/2uum25v. CAF, cancer-associated fibroblast; DC, dendritic cell; ECM, extracellular matrix; GPR81, G protein-coupled receptor 81; ICI, immune-checkpoint inhibitor; MCT, monocarboxylate transporter; MDSC, myeloid-derived suppressor cell; TAM, tumor-associated macrophage; Treg, regulatory T cell

Combination therapy should be selected according to the dominant biological barrier. Lactate-high, MCT4-high tumors with preserved immune-cell access may be candidates for lactate-lowering or transport-blocking strategies combined with checkpoint inhibition. Tumors with strong CAF/ECM exclusion may require stromal remodeling before metabolic correction can improve T-cell access. Tumors with suppressed antigen-presentation or persistent transcriptional silencing may require short-course epigenetic priming, although a lactylation-specific mechanism must be demonstrated before such combinations are described as lactylation-targeted therapy. A conceptual framework linking spatial immune exclusion to layered therapeutic intervention is shown in Fig. 4.

Predictive biomarkers should integrate tumor subtype, extracellular lactate or pH, LDHA and MCT1/MCT4 expression, hypoxia, GPR81 expression, immune-cell composition, and spatial localization. Lactylation markers such as H3K18la, H4K12la, or defined non-histone sites may provide additional information, but antibody-based measurements should be validated by orthogonal methods. On-treatment sampling will also be required to distinguish incomplete target inhibition, metabolic compensation, persistent stromal exclusion, and failure to reverse established immune states.

## Conclusion

Lactate shapes the breast cancer TME through mechanisms that extend beyond its role as a glycolytic end product. Hypoxia-driven production, MCT1/MCT4-dependent transport, extracellular acidification, metabolic competition, and GPR81/HCAR1 signaling can rapidly impair cytotoxic lymphocytes and dendritic cells while favoring regulatory, myeloid, and stromal programs. These effects provide a well-supported basis for acute metabolic suppression and progressive lineage remodeling.

Lactylation adds a potential epigenetic and post-translational layer to this framework. Direct breast cancer studies increasingly support roles for histone and non-histone lactylation in tumor-cell survival, metabolic adaptation, invasion, and metastasis. However, breast cancer-specific evidence linking defined lactylation sites to immune escape remains limited and is weighted toward TNBC. Lactylation should therefore be regarded as one candidate mechanism of durable state fixation rather than a universal explanation for lactate-associated phenotypes.

Future studies should combine quantitative site-resolved lactylation measurements with functional perturbation, isotope tracing, single-cell multi-omics, spatial epigenomic profiling, and patient-derived models. These approaches will be needed to determine which cell populations are directly modified, whether lactylation persists after metabolic pressure is removed, and which tumors are genuinely dependent on lactate-driven epigenetic regulation. Therapeutically, targeting lactate production, transport, sensing, or downstream chromatin regulation may support rational combination strategies, but clinical development will require subtype-specific biomarkers, careful treatment sequencing, and clear separation of lactylation-specific effects from broader metabolic and epigenetic responses.

## Data Availability

No datasets were generated or analysed during the current study.

## References

[1] Harbeck N, Gnant M. Breast cancer. Lancet. 2017;389(10074):1134–50. 10.1016/S0140-6736(16)31891-8 27865536

[2] Qiao Y, Liu Y, Ran R, Zhou Y, Gong J, Liu L, et al. Lactate metabolism and lactylation in breast cancer: mechanisms and implications. Cancer Metastasis Rev. 2025;44(2):48. 10.1007/s10555-025-10264-4 PMC12037681 40295451

[3] Zou J, Mai C, Lin Z, Zhou J, Lai G. Targeting metabolism of breast cancer and its implications in T cell immunotherapy. Front Immunol. 2024;15:1381970. 10.3389/fimmu.2024.1381970 PMC11045902 38680483

[4] Pavlova NN, Thompson CB. The Emerging Hallmarks of Cancer Metabolism. Cell Metab. 2016;23(1):27–47. 10.1016/j.cmet.2015.12.006 PMC4715268 26771115

[5] Brand A, Singer K, Koehl GE, Kolitzus M, Schoenhammer G, Thiel A, et al. LDHA-Associated Lactic Acid Production Blunts Tumor Immunosurveillance by T and NK Cells. Cell Metab. 2016;24(5):657–71. 10.1016/j.cmet.2016.08.011 27641098

[6] Huang L, Chen X, Yan M, Xiang Z, Wu J. Lactate and lactylation in breast cancer: current understanding and therapeutic opportunities. Cancer Biol Med. 2025;22(7):789–805. 10.20892/j.issn.2095-3941.2025.0173 PMC12302272 40671416

[7] Wang JX, Choi SYC, Niu X, Kang N, Xue H, Killam J, et al. Lactic acid and an acidic tumor microenvironment suppress anticancer immunity. Int J Mol Sci. 2020;21(21):8363. 10.3390/ijms21218363 PMC7664620 33171818

[8] Calcinotto A, Filipazzi P, Grioni M, Iero M, De Milito A, Ricupito A, et al. Modulation of microenvironment acidity reverses anergy in human and murine tumor-infiltrating T lymphocytes. Cancer Res. 2012;72(11):2746–56. 10.1158/0008-5472.CAN-11-1272 22593198

[9] Morrot A, da Fonseca LM, Salustiano EJ, Gentile LB, Conde L, Filardy AA, et al. Metabolic Symbiosis and Immunomodulation: How Tumor Cell-Derived Lactate May Disturb Innate and Adaptive Immune Responses. Front Oncol. 2018;8:81. 10.3389/fonc.2018.00081 PMC5876249 29629338

[10] Brooks GA. The Science and Translation of Lactate Shuttle Theory. Cell Metab. 2018;27(4):757–85. 10.1016/j.cmet.2018.03.008 29617642

[11] Brown TP, Bhattacharjee P, Ramachandran S, Sivaprakasam S, Ristic B, Sikder MOF, et al. The lactate receptor GPR81 promotes breast cancer growth via a paracrine mechanism involving antigen-presenting cells in the tumor microenvironment. Oncogene. 2020;39(16):3292–304. 10.1038/s41388-020-1216-5 32071396

[12] Ishihara S, Hata K, Hirose K, Okui T, Toyosawa S, Uzawa N, et al. The lactate sensor GPR81 regulates glycolysis and tumor growth of breast cancer. Sci Rep. 2022;12(1):6261. 10.1038/s41598-022-10143-w PMC9012857 35428832

[13] Certo M, Llibre A, Lee W, Mauro C. Understanding lactate sensing and signalling. Trends Endocrinol Metab. 2022;33(10):722–35. 10.1016/j.tem.2022.07.004 35999109

[14] Zhang D, Tang Z, Huang H, Zhou G, Cui C, Weng Y, et al. Metabolic regulation of gene expression by histone lactylation. Nature. 2019;574(7779):575–80. 10.1038/s41586-019-1678-1 PMC6818755 31645732

[15] Wu X, Tao WA. Uncovering ubiquitous protein lactylation. Nat Methods. 2022;19(7):793–4. 10.1038/s41592-022-01536-w 35761068

[16] Li H, Sun L, Gao P, Hu H. Lactylation in cancer: Current understanding and challenges. Cancer Cell. 2024;42(11):1803–7. 10.1016/j.ccell.2024.09.006 39393355

[17] Llibre A, Kucuk S, Gope A, Certo M, Mauro C, Lactate. A key regulator of the immune response. Immunity. 2025;58(3):535–54. 10.1016/j.immuni.2025.02.008 40073846

[18] Lundo K, Dmytriyeva O, Spohr L, Goncalves-Alves E, Yao J, Blasco LP, et al. Lactate receptor GPR81 drives breast cancer growth and invasiveness through regulation of ECM properties and Notch ligand DLL4. BMC Cancer. 2023;23(1):1136. 10.1186/s12885-023-11631-6 PMC10666402 37993804

[19] Watson MJ, Vignali PDA, Mullett SJ, Overacre-Delgoffe AE, Peralta RM, Grebinoski S, et al. Metabolic support of tumour-infiltrating regulatory T cells by lactic acid. Nature. 2021;591(7851):645–51. 10.1038/s41586-020-03045-2 PMC7990682 33589820

[20] Mu X, Shi W, Xu Y, Xu C, Zhao T, Geng B, et al. Tumor-derived lactate induces M2 macrophage polarization via the activation of the ERK/STAT3 signaling pathway in breast cancer. Cell Cycle. 2018;17(4):428–38. 10.1080/15384101.2018.1444305 PMC5927648 29468929

[21] Plebanek MP, Xue Y, Nguyen YV, DeVito NC, Wang X, Holtzhausen A, et al. A lactate-SREBP2 signaling axis drives tolerogenic dendritic cell maturation and promotes cancer progression. Sci Immunol. 2024;9(95):eadi4191. 10.1126/sciimmunol.adi4191 PMC11926670 38728412

[22] Cappellesso F, Mazzone M, Virga F. Acid affairs in anti-tumour immunity. Cancer Cell Int. 2024;24(1):354. 10.1186/s12935-024-03520-0 PMC11514911 39465367

[23] Rahman MA, Yadab MK, Ali MM. Emerging Role of Extracellular pH in Tumor Microenvironment as a Therapeutic Target for Cancer Immunotherapy. Cells. 2024;13:22. 10.3390/cells13221924 PMC11592846 39594672

[24] Baltazar F, Afonso J, Costa M, Granja S. Lactate Beyond a Waste Metabolite: Metabolic Affairs and Signaling in Malignancy. Front Oncol. 2020;10:231. 10.3389/fonc.2020.00231 PMC7093491 32257942

[25] Semenza GL. Hypoxia-inducible factor 1 and cancer pathogenesis. IUBMB Life. 2008;60(9):591–7. 10.1002/iub.93 18506846

[26] Ippolito L, Morandi A, Giannoni E, Chiarugi P. Lactate: A Metabolic Driver in the Tumour Landscape. Trends Biochem Sci. 2019;44(2):153–66. 10.1016/j.tibs.2018.10.011 30473428

[27] Hussien R, Brooks GA. Mitochondrial and plasma membrane lactate transporter and lactate dehydrogenase isoform expression in breast cancer cell lines. Physiol Genomics. 2011;43(5):255–64. 10.1152/physiolgenomics.00177.2010 PMC3068517 21177384

[28] Hong CS, Graham NA, Gu W, Espindola Camacho C, Mah V, Maresh EL, et al. MCT1 Modulates Cancer Cell Pyruvate Export and Growth of Tumors that Co-express MCT1 and MCT4. Cell Rep. 2016;14(7):1590–601. 10.1016/j.celrep.2016.01.057 PMC4816454 26876179

[29] Baenke F, Dubuis S, Brault C, Weigelt B, Dankworth B, Griffiths B, et al. Functional screening identifies MCT4 as a key regulator of breast cancer cell metabolism and survival. J Pathol. 2015;237(2):152–65. 10.1002/path.4562 25965974

[30] Jin L, Guo Y, Chen J, Wen Z, Jiang Y, Qian J. Lactate receptor HCAR1 regulates cell growth, metastasis and maintenance of cancer–specific energy metabolism in breast cancer cells. Mol Med Rep. 2022;26(2):268. 10.3892/mmr.2022.12784 PMC9260879 35775372

[31] Guo S, Zhou J, Lou P, Weng L, Ye X, Guo J, et al. Potentiated effects of lactate receptor GPR81 on immune microenvironment in breast cancer. Mol Carcinog. 2023;62(9):1369–77. 10.1002/mc.23582 37249360

[32] Xia L, Oyang L, Lin J, Tan S, Han Y, Wu N, et al. The cancer metabolic reprogramming and immune response. Mol Cancer. 2021;20(1):28. 10.1186/s12943-021-01316-8 PMC7863491 33546704

[33] Faubert B, Solmonson A, DeBerardinis RJ. Metabolic reprogramming and cancer progression. Science. 2020;368(6487):eaaw5473. 10.1126/science.aaw5473 PMC7227780 32273439

[34] Peralta RM, Xie B, Lontos K, Nieves-Rosado H, Spahr K, Joshi S, et al. Dysfunction of exhausted T cells is enforced by MCT11-mediated lactate metabolism. Nat Immunol. 2024;25(12):2297–307. 10.1038/s41590-024-01999-3 PMC11588660 39516648

[35] Babl N, Decking SM, Voll F, Althammer M, Sala-Hojman A, Ferretti R, et al. MCT4 blockade increases the efficacy of immune checkpoint blockade. J Immunother Cancer. 2023;11(10):e007349. 10.1136/jitc-2023-007349 PMC10603342 37880183

[36] Ding R, Yu X, Hu Z, Dong Y, Huang H, Zhang Y, et al. Lactate modulates RNA splicing to promote CTLA-4 expression in tumor-infiltrating regulatory T cells. Immunity. 2024;57(3):528–40. e6. 10.1016/j.immuni.2024.01.019 38417442

[37] Xue Q, Peng W, Zhang S, Wei X, Ye L, Wang Z, et al. Lactylation-driven TNFR2 expression in regulatory T cells promotes the progression of malignant pleural effusion. J Immunother Cancer. 2024;12(12):e010040. 10.1136/jitc-2024-010040 PMC11683941 39721754

[38] Kumagai S, Koyama S, Itahashi K, Tanegashima T, Lin YT, Togashi Y, et al. Lactic acid promotes PD-1 expression in regulatory T cells in highly glycolytic tumor microenvironments. Cancer Cell. 2022;40(2):201–18. e9. 10.1016/j.ccell.2022.01.001 35090594

[39] Shi Y, Yasui M, Hara-Chikuma M. AQP9 transports lactate in tumor-associated macrophages to stimulate an M2-like polarization that promotes colon cancer progression. Biochem Biophys Rep. 2022;31:101317. 10.1016/j.bbrep.2022.101317 PMC9372591 35967760

[40] Noe JT, Rendon BE, Geller AE, Conroy LR, Morrissey SM, Young LEA, et al. Lactate supports a metabolic-epigenetic link in macrophage polarization. Sci Adv. 2021;7(46):eabi8602. 10.1126/sciadv.abi8602 PMC8589316 34767443

[41] Fang X, Zhao P, Gao S, Liu D, Zhang S, Shan M, et al. Lactate induces tumor-associated macrophage polarization independent of mitochondrial pyruvate carrier-mediated metabolism. Int J Biol Macromol. 2023;237:123810. 10.1016/j.ijbiomac.2023.123810 36868333

[42] Jiang M, Wang Y, Zhao X, Yu J. From metabolic byproduct to immune modulator: the role of lactate in tumor immune escape. Front Immunol. 2024;15:1492050. 10.3389/fimmu.2024.1492050 PMC11625744 39654883

[43] Pavlova A, Mushii O, Bazas V, Karacharova I, Zadvornyi T, Lukianova N. Tumor-Associated Macrophages: Relationship with Clinical Status of Patients and Molecular Biological Features of Breast Cancer. Exp Oncol. 2025;47(2):197–206. 10.15407/exp-oncology.2025.02.197 41058461

[44] Zong Z, Xie F, Wang S, Wu X, Zhang Z, Yang B, et al. Alanyl-tRNA synthetase, AARS1, is a lactate sensor and lactyltransferase that lactylates p53 and contributes to tumorigenesis. Cell. 2024;187(10):2375–e9233. 10.1016/j.cell.2024.04.002 38653238

[45] Iozzo M, Pardella E, Giannoni E, Chiarugi P. The role of protein lactylation: A kaleidoscopic post-translational modification in cancer. Mol Cell. 2025;85(7):1263–79. 10.1016/j.molcel.2025.02.011 40073861

[46] Xiao S, Zhang S, Sun K, Huang Q, Li Q, Hu C. Lactate and lactylation: molecular insights into histone and non-histone lactylation in tumor progression, tumor immune microenvironment, and therapeutic strategies. Biomark Res. 2025;13(1):134. 10.1186/s40364-025-00849-0 PMC12553170 41137039

[47] Hu Y, He Z, Li Z, Wang Y, Wu N, Sun H, et al. Lactylation: the novel histone modification influence on gene expression, protein function, and disease. Clin Epigenetics. 2024;16(1):72. 10.1186/s13148-024-01682-2 PMC11138093 38812044

[48] Gaffney DO, Jennings EQ, Anderson CC, Marentette JO, Shi T, Schou Oxvig AM, et al. Non-enzymatic Lysine Lactoylation of Glycolytic Enzymes. Cell Chem Biol. 2020;27(2):206–13. e6. 10.1016/j.chembiol.2019.11.005 PMC7395678 31767537

[49] Zhang D, Gao J, Zhu Z, Mao Q, Xu Z, Singh PK, et al. Lysine L-lactylation is the dominant lactylation isomer induced by glycolysis. Nat Chem Biol. 2025;21(1):91–9. 10.1038/s41589-024-01680-8 PMC11666458 39030363

[50] Niu Z, Chen C, Wang S, Lu C, Wu Z, Wang A, et al. HBO1 catalyzes lysine lactylation and mediates histone H3K9la to regulate gene transcription. Nat Commun. 2024;15(1):3561. 10.1038/s41467-024-47900-6 PMC11053077 38670996

[51] Xie B, Zhang M, Li J, Cui J, Zhang P, Liu F, et al. KAT8-catalyzed lactylation promotes eEF1A2-mediated protein synthesis and colorectal carcinogenesis. Proc Natl Acad Sci U S A. 2024;121(8):e2314128121. 10.1073/pnas.2314128121 PMC10895275 38359291

[52] Zhang ZL, Ren ST, Yang WJ, Xu XW, Zhao SM, Fang KF, et al. AARS2-catalyzed lactylation induces follicle development and premature ovarian insufficiency. Cell Death Discov. 2025;11(1):209. 10.1038/s41420-025-02501-0 PMC12041370 40301335

[53] Wang S, Lv H, Zhou F. AARS1 and AARS2, the L-lactate sensors and universal lactyltransferases. Sci Bull (Beijing). 2025;70(10):1547–9. 10.1016/j.scib.2024.11.049 39730223

[54] Gonzatti MB, Hintzen JCJ, Sharma I, Najar MA, Tsusaka T, Marcinkiewicz MM, et al. Class I histone deacetylases catalyze lysine lactylation. J Biol Chem. 2025;301(10):110602. 10.1016/j.jbc.2025.110602 PMC12624779 40835008

[55] Moreno-Yruela C, Zhang D, Wei W, Baek M, Liu W, Gao J, et al. Class I histone deacetylases (HDAC1-3) are histone lysine delactylases. Sci Adv. 2022;8(3):eabi6696. 10.1126/sciadv.abi6696 PMC8769552 35044827

[56] Du R, Gao Y, Yan C, Ren X, Qi S, Liu G, et al. Sirtuin 1/sirtuin 3 are robust lysine delactylases and sirtuin 1-mediated delactylation regulates glycolysis. iScience. 2024;27(10):110911. 10.1016/j.isci.2024.110911 PMC11440250 39351192

[57] Hu X, Huang X, Yang Y, Sun Y, Zhao Y, Zhang Z, et al. Dux activates metabolism-lactylation-MET network during early iPSC reprogramming with Brg1 as the histone lactylation reader. Nucleic Acids Res. 2024;52(10):5529–48. 10.1093/nar/gkae183 PMC11162783 38512058

[58] Nunez R, Sidlowski PFW, Steen EA, Wynia-Smith SL, Sprague DJ, Keyes RF, et al. The TRIM33 Bromodomain Recognizes Histone Lysine Lactylation. ACS Chem Biol. 2024;19(12):2418–28. 10.1021/acschembio.4c00248 PMC11706526 39556662

[59] Zhai G, Niu Z, Jiang Z, Zhao F, Wang S, Chen C, et al. DPF2 reads histone lactylation to drive transcription and tumorigenesis. Proc Natl Acad Sci U S A. 2024;121(50):e2421496121. 10.1073/pnas.2421496121 PMC11648877 39636855

[60] Li J, Chen Z, Jin M, Gu X, Wang Y, Huang G, et al. Histone H4K12 lactylation promotes malignancy progression in triple-negative breast cancer through SLFN5 downregulation. Cell Signal. 2024;124:111468. 10.1016/j.cellsig.2024.111468 39395526

[61] Cui Z, Li Y, Lin Y, Zheng C, Luo L, Hu D, et al. Lactylproteome analysis indicates histone H4K12 lactylation as a novel biomarker in triple-negative breast cancer. Front Endocrinol (Lausanne). 2024;15:1328679. 10.3389/fendo.2024.1328679 PMC11109423 38779451

[62] Xu Y, Meng W, Dai Y, Xu L, Ding N, Zhang J, et al. Anaerobic metabolism promotes breast cancer survival via Histone-3 Lysine-18 lactylation mediating PPARD axis. Cell Death Discov. 2025;11(1):54. 10.1038/s41420-025-02334-x PMC11807217 39922804

[63] Pandkar MR, Sinha S, Samaiya A, Shukla S. Oncometabolite lactate enhances breast cancer progression by orchestrating histone lactylation-dependent c-Myc expression. Transl Oncol. 2023;37:101758. 10.1016/j.tranon.2023.101758 PMC10425713 37572497

[64] Hou X, Ouyang J, Tang L, Wu P, Deng X, Yan Q, et al. KCNK1 promotes proliferation and metastasis of breast cancer cells by activating lactate dehydrogenase A (LDHA) and up-regulating H3K18 lactylation. PLoS Biol. 2024;22(6):e3002666. 10.1371/journal.pbio.3002666 PMC11192366 38905316

[65] Zhao Z, Jia W, Li Z, Shi S, Zhou J, Ning C, et al. Targeting the SIN1 mediated TTK/LDHA-H3K18la-GLUT3 axis disrupts metabolic-epigenetic crosstalk and suppresses progression in hyperglycolytic breast cancer. Cancer Lett. 2026;644:218328. 10.1016/j.canlet.2026.218328 41707978

[66] Liu S, Bai Z, Qiao Y, Ge Y, Qu F, Li M, et al. LDHA-mediated CREB1 lactylation promotes breast cancer brain metastasis by transcriptionally activating cytoskeletal remodeling. Neuro Oncol. 2026. 10.1093/neuonc/noag114 PMC13437888 42166760

[67] Chen J, Hao Y, Feng Y, Dai Y, Shi H, Zhu L. Lactylation-mediated remodelling of the breast cancer microenvironment: single-cell multidimensional analysis and prognostic model construction. Front Immunol. 2026;17:1747043. 10.3389/fimmu.2026.1747043 PMC13212231 42212139

[68] Ma Z, Yang J, Jia W, Li L, Li Y, Hu J, et al. Histone lactylation-driven B7-H3 expression promotes tumor immune evasion. Theranostics. 2025;15(6):2338–59. 10.7150/thno.105947 PMC11840737 39990209

[69] Hu X, Huang Z, Li L. LDHB Mediates Histone Lactylation to Activate PD-L1 and Promote Ovarian Cancer Immune Escape. Cancer Invest. 2025;43(1):70–9. 10.1080/07357907.2024.2430283 39587817

[70] Huang ZW, Zhang XN, Zhang L, Liu LL, Zhang JW, Sun YX, et al. STAT5 promotes PD-L1 expression by facilitating histone lactylation to drive immunosuppression in acute myeloid leukemia. Signal Transduct Target Ther. 2023;8(1):391. 10.1038/s41392-023-01605-2 PMC10542808 37777506

[71] Sun K, Zhang X, Shi J, Huang J, Wang S, Li X, et al. Elevated protein lactylation promotes immunosuppressive microenvironment and therapeutic resistance in pancreatic ductal adenocarcinoma. J Clin Invest. 2025. 10.1172/JCI187024 PMC11957693 39883522

[72] Wang R, Li C, Cheng Z, Li M, Shi J, Zhang Z, et al. H3K9 lactylation in malignant cells facilitates CD8(+) T cell dysfunction and poor immunotherapy response. Cell Rep. 2024;43(9):114686. 10.1016/j.celrep.2024.114686 39216002

[73] Cai Y, Li X, Zhu S, Wu H, Nie J, Huang F. Lactate represses MHC-I antigen presentation via H4K5 lactylation-PRC2 in small cell lung cancer subtype A. Int Immunopharmacol. 2026;170:116125. 10.1016/j.intimp.2025.116125 41468795

[74] Donnellan L, Young C, Seidel JD, Peake B, Lau D, Deo P, et al. An Investigation into the Differentiation of Lactyllysine and Carboxyethyllysine Peptide Modifications by Liquid Chromatography/Mass Spectrometry. J Proteome Res. 2026;25(3):1601–10. 10.1021/acs.jproteome.5c00969 PMC12974333 41679736

[75] Manessier J, Hijazi H, Vizzini L, Brugiere S, Courcon M, Masselon C, et al. Both L-Lactyl and D-Lactyl Enantiomers Modify Histones in Mouse Testis. Mol Cell Proteom. 2026;25(7):101601. 10.1016/j.mcpro.2026.101601 PMC13380202 42285509

[76] Stine ZE, Schug ZT, Salvino JM, Dang CV. Targeting cancer metabolism in the era of precision oncology. Nat Rev Drug Discov. 2022;21(2):141–62. 10.1038/s41573-021-00339-6 PMC8641543 34862480

[77] Raez LE, Papadopoulos K, Ricart AD, Chiorean EG, Dipaola RS, Stein MN, et al. A phase I dose-escalation trial of 2-deoxy-D-glucose alone or combined with docetaxel in patients with advanced solid tumors. Cancer Chemother Pharmacol. 2013;71(2):523–30. 10.1007/s00280-012-2045-1 23228990

[78] Halford S, Veal GJ, Wedge SR, Payne GS, Bacon CM, Sloan P, et al. A Phase I Dose-escalation Study of AZD3965, an Oral Monocarboxylate Transporter 1 Inhibitor, in Patients with Advanced Cancer. Clin cancer research: official J Am Association Cancer Res. 2023;29(8):1429–39. 10.1158/1078-0432.CCR-22-2263 PMC7614436 36652553

[79] Mohammad Nezhady MA, Modaresinejad M, Zia A, Chemtob S. Versatile lactate signaling via HCAR1: a multifaceted GPCR involved in many biological processes. Am J Physiol Cell Physiol. 2023;325(6):C1502–15. 10.1152/ajpcell.00346.2023 37899751

[80] Papadatos-Pastos D, Yuan W, Pal A, Crespo M, Ferreira A, Gurel B, et al. Phase 1, dose-escalation study of guadecitabine (SGI-110) in combination with pembrolizumab in patients with solid tumors. J Immunother Cancer. 2022. 10.1158/1538-7445.AM2020-CT129 PMC9240883 35717027

[81] Jang HJ, Hostetter G, Macfarlane AW, Madaj Z, Ross EA, Hinoue T, et al. A Phase II Trial of Guadecitabine plus Atezolizumab in Metastatic Urothelial Carcinoma Progressing after Initial Immune Checkpoint Inhibitor Therapy. Clin cancer research: official J Am Association Cancer Res. 2023;29(11):2052–65. 10.1158/1078-0432.CCR-22-3642 PMC10233355 36928921

